# Cortico-Striatal Functional Connectivity Reflects Changes in Subjective Energy and Tiredness

**DOI:** 10.1093/geroni/igab046.1434

**Published:** 2021-12-17

**Authors:** James Hengenius, Rebecca Ehrenkranz, Theodore Huppert, Caterina Rosano

**Affiliations:** 1 University of Pittsburgh, Pittsburgh, Pennsylvania, United States; 2 University of Pittsburgh Graduate School of Public Health, Pittsburgh, Pennsylvania, United States

## Abstract

Subjective feelings of energy and tiredness may reflect different neural processes. Functional connectivity (FC) was measured in 272 HealthABC participants via resting state functional MRI in striatal-associative, striatal-limbic and striatal-sensorimotor networks. Subjective energy level (scored 1-10) and tiredness (tired/not-tired) during the prior month were collected via self-report from year 2 to year 10 (mean energy follow-up=8 years, tiredness follow-up=7 years). Participants who never reported being tired during follow-up (N=119) had significantly lower FC in the striatal-limbic network (mean difference [95%CI]: -0.055 [-0.1020,-0.00879], p=0.02). Participants with stable energy level over time (N=94, defined as decline <1.0 SD below the mean) had significantly higher FC in the striatal-associative network (mean difference [95% CI]: 0.041 [0.00192,0.0807], p=0.04). Associations were similar when adjusted for brain atrophy, demographics, and education. Although based on subjective measures, the distinct spatial patterns of these associations support our hypothesis that neural basis of energy and fatigue may differ.

